# Perspective on acupuncture treatment for diabetic peripheral neuropathy: A perspective

**DOI:** 10.1097/MD.0000000000042646

**Published:** 2025-06-06

**Authors:** Lin Wang, Li-min Wang

**Affiliations:** a Department of Endocrinology, Xi’an Gaoxin Hospital, Xi’an, China; b Department of Clinical Laboratory, Shaanxi Province Hospital of Traditional Chinese Medicine, Xi’an, China.

**Keywords:** acupuncture, diabetic peripheral neuropathy, perspective

## Abstract

Acupuncture is increasingly recognized for its potential in managing diabetic peripheral neuropathy (DPN). This condition severely impacts quality of life by inducing symptoms such as pain and sensory loss, and, in severe cases, leading to amputation. Research indicates that acupuncture may alleviate these symptoms through mechanisms that include enhancing neural blood flow, promoting nerve regeneration, and modulating glucose metabolism. Despite these promising findings, the variability in acupuncture’s effectiveness, along with the methodological limitations of existing studies-such as small sample sizes and lack of standardization-pose challenges. Future research should therefore focus on conducting large-scale, high-quality trials that could elucidate the specific mechanisms by which acupuncture benefits DPN and confirm its long-term efficacy. This would facilitate acupuncture’s integration into comprehensive DPN management strategies, potentially improving outcomes by combining it with pharmacological and other therapies. Optimizing treatment protocols and personalizing acupuncture based on individual patient profiles could further enhance its effectiveness and integration into clinical practice.

## 1. Introduction

### 1.1. Epidemiological background of diabetic peripheral neuropathy (DPN)

Diabetes mellitus represents a major global health challenge, with the International Diabetes Federation reporting a continuous increase in the prevalence among adults worldwide, projected to reach 700 million by 2045.^[1]^ Diabetes is not only prevalent but also associated with numerous complications. Diabetic peripheral neuropathy (DPN) is one of the most common chronic complications, affecting approximately 50% of individuals with diabetes.^[1–3]^

The clinical manifestations of DPN include sensory loss, pain, tingling, or burning sensations in the hands and feet, significantly impacting patients’ quality of life and daily activities.^[2,3]^ As the condition progresses, patients may experience complete sensory loss, increasing the risk of injuries and, in severe cases, necessitating amputation.^[2,3]^ Thus, effective management of DPN is crucial for enhancing the quality of life of patients with diabetes.^[3,4]^

### 1.2. Traditional theory and practical background of acupuncture

Acupuncture is a significant branch of traditional Chinese medicine (TCM) with thousands of years of therapeutic history.^[5,6]^ According to the fundamental theories of TCM, health is closely linked to the flow of “*Qi*,” the balance of *Yin* and *Yang*, and the functional states of the organs.^[5,6]^ Acupuncture achieves therapeutic effects by stimulating specific points on the body to regulate *Qi* and *Blood* circulation, thereby restoring *Yin* and *Yang* balance and curing diseases.^[5,6]^

In the context of DPN, specific acupoints associated with the *Liver*, *Spleen*, and *Kidney* meridians are frequently selected due to their putative roles in nerve function and microcirculation.^[7]^ Clinical applications often emphasize acupoints such as Zusanli (ST36), Sanyinjiao (SP6), and Yongquan (KI1), which have been reported to enhance peripheral nerve conductivity and improve local blood flow.^[7]^ Research suggests that stimulation of these points may contribute to neuroprotection by increasing nerve growth factor expression and modulating inflammatory pathways relevant to neuropathic pain.^[8]^

In treating neurological disorders, acupuncture is utilized to enhance neural function, promote blood circulation, and facilitate the repair of injured nerves.^[9]^ Both historical records and modern research indicate that acupuncture can effectively treat various neurological disorders, including Parkinson’s disease, post sequelae, and neuropathic pain.^[9–12]^ Recent scientific investigations have begun to unveil potential mechanisms of acupuncture in neural regulation, such as promoting the release of endogenous neurotrophic factors, which are vital for neuroprotection and regeneration.^[9]^

Although the precise mechanisms of acupuncture remain partially understood, its potential in alleviating symptoms of DPN has been substantiated by multiple clinical studies. These studies demonstrate that acupuncture can significantly improve symptoms such as pain and sensory abnormalities in DPN patients, thereby enhancing their quality of life. However, the therapeutic outcomes of acupuncture vary among individuals, and further research is needed to elucidate its long-term effects and optimal treatment protocols.

## 2. Search strategy and study selection

A systematic literature search was conducted using the China National Knowledge Infrastructure and PubMed databases to identify relevant studies on acupuncture treatment for DPN. The search covered all available publications from the inception of these databases through October 1, 2024. The search strategy employed a combination of controlled vocabulary and free-text keywords, including “acupuncture” and “DPN,” ensuring a comprehensive retrieval of pertinent literature. Only studies published in Chinese and English were included to incorporate both domestic and international research perspectives.

To maintain methodological rigor, predefined eligibility criteria were applied. Only randomized controlled trials, controlled trials, and observational studies published in peer-reviewed journals were considered. Studies were required to focus specifically on the clinical efficacy and therapeutic mechanisms of acupuncture in DPN management. Articles were excluded if they did not directly investigate acupuncture for DPN, were preclinical or mechanistic studies, or were duplicates of previously identified publications.

The initial search identified 622 articles. After screening based on title and abstract, 532 studies were excluded due to irrelevance, duplication, or failure to meet the inclusion criteria. A total of 90 full-text articles underwent detailed review. Following a rigorous assessment of study design, data quality, and relevance, 9 studies met all inclusion criteria and were included in this study (Table 1).

**Table 1:** Summary of clinical studies on acupuncture for patients with diabetic peripheral neuropathy.

Study ref	Publication type	Disease	Sample size	Treatment	Main findings
Hoerder et al^[13]^	Randomized controlled trial	T2DPN	62	Acupuncture vs waiting list	Acupuncture may help reduce neurological deficits in T2DPN without serious adverse events
Shu et al^[14]^	Randomized controlled trial	T2DPN	60	Acupuncture	Acupuncture effectively enhances neurological function and alleviates clinical symptoms in T2DPN patients
Meyer-Hamme et al^[15]^	Randomized controlled trial	T2DPN	180 (172 completed)	Needle and laser acupuncture vs placebo	Classical needle acupuncture significantly improved DPN. Enhanced nerve conduction study values suggest possible structural neuroregeneration post
Ma et al^[16]^	Randomized controlled trial	DPN	64	Warming acupuncture vs acupuncture	Warming acupuncture showed better symptom relief than conventional acupuncture in treating DPN with yang deficiency and cold coagulation
Chen et al^[17]^	Randomized controlled trial	DPN	63	Acupuncture	Penetrating acupuncture improved lower limb nerve conduction in DPN patients more than mecobalamin, potentially due to electroacupuncture use
Zhao et al^[18]^	Randomized controlled trial	DPN	60	Acupuncture and oral methycobal vs basic treatment	Acupuncture can alleviate neurological symptoms, reduce blood viscosity, and enhance nerve conduction velocity in patients with DPN
Bailey et al^[19]^	Observational study	T2DPN	25 (19 completed)	Acupuncture	Nineteen of 25 participants experienced significant relief in several DPN symptoms with acupuncture, though improvements in lancinating pain and laser Doppler fluxmetry measures were minimal
Tong et al^[20]^	Controlled trial	DPN	63	Acupuncture vs sham acupuncture	Acupuncture significantly outperformed sham treatments in alleviating numbness, spontaneous pain, rigidity, and temperature perception changes in DPN, suggesting its potential clinical utility
Zhang et al^[21]^	Randomized controlled trial	DPN	65	Acupuncture vs oral inositol	Acupuncture has shown promising results in treatinsg DPN

DPN = diabetic peripheral neuropathy, T2DPN = type 2 DNP.

## 3. Research advances of acupuncture for DPN

### 3.1. Mechanisms of acupuncture in the treatment of DPN

**Figure 1.**
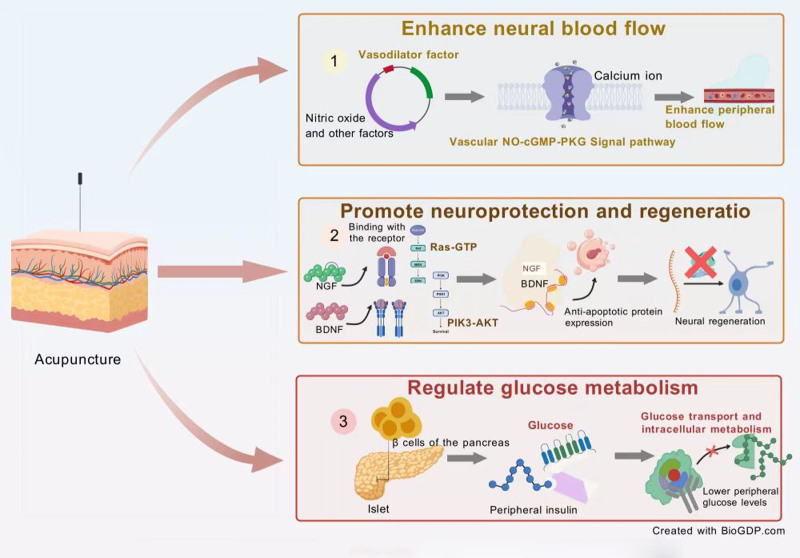
Possible mechanism of acupuncture for diabetic peripheral neuropathy. BDNF = brain-derived neurotrophic factor, NGF = nerve growth factor, NO-cGMP-PKG = nitric oxide-cyclic guanosine monophosphate-protein kinase G, PIK3-AKT = phosphoinositide 3-kinase-protein kinase B, Ras-GTP = Ras guanosine triphosphate.

Acupuncture has gained attention in the management of DPN due to its potential to mitigate neuropathic pain and aid in nerve function recovery.^[22–25]^ The therapeutic mechanisms attributed to acupuncture in treating DPN can be delineated into 3 primary areas: enhancement of neural blood flow, promotion of neuroprotection and regeneration, and regulation of glucose metabolism^[22–25]^ (Fig. 1).

#### 3.1.1. Enhancement of neural blood flow

Acupuncture is hypothesized to facilitate the release of vasodilators, notably nitric oxide, which significantly contributes to blood vessel dilation and improved circulation.^[26,27]^ Enhanced peripheral blood flow can help alleviate the ischemic conditions frequently observed in DPN, thus reducing nerve damage and supporting improved nerve function.^[26,27]^ Clinical evidence suggests that targeting acupuncture points along affected nerve pathways can boost microcirculation specifically in those regions, directly benefiting nerves impaired by diabetic neuropathy.

#### 3.1.2. Promotion of neuroprotection and regeneration

Emerging research indicates that acupuncture can modulate the expression of neurotrophic factors, critical for neuronal survival and repair.^[22,23]^ Studies have documented increases in nerve growth factor and brain-derived neurotrophic factor following acupuncture treatment. These neurotrophins are essential for maintaining neuronal integrity and can potentially counteract the deleterious effects of diabetes on peripheral nerves. By enhancing the health and regeneration capabilities of nerve cells, acupuncture may restore neural functions compromised by DPN.

#### 3.1.3. Regulation of glucose metabolism

Acupuncture also appears to influence glucose metabolism, a key concern for individuals with diabetes.^[28–31]^ Although the direct mechanisms remain under exploration, initial research points to acupuncture’s ability to improve insulin sensitivity and increase glucose uptake in peripheral tissues.^[28–30]^ This action may lead to improved glycemic control, a critical factor in slowing the progression of diabetes-related complications, including neuropathy.

In summary, acupuncture’s role in treating DPN involves complex physiological processes that collectively enhance neural blood flow, stimulate neuroprotection and regeneration, and potentially regulate glucose metabolism. These interconnected mechanisms provide a compelling case for acupuncture as a complementary intervention in the management of diabetic neuropathy, offering symptomatic relief and a possible reversal of nerve injury.

### 3.2. Review and analysis of clinical studies

#### 3.2.1. Review of recent key clinical trials

In recent years, numerous clinical trials have assessed the efficacy of acupuncture in treating DPN, especially type 2 DPN (T2DPN). For instance, the study by Hoerder et al demonstrated that acupuncture could significantly reduce neurological deficits such as numbness in patients with T2DPN^[13]^ (Table 1). Another study by Shu et al focused on the effects of acupuncture at specific points such as the xing-spring and shu-stream, finding significant improvements in neurological function and clinical symptoms^[14]^ (Table 1).

#### 3.2.2. Methodological evaluation: trial design, sample size, intervention measures, and outcome metrics

These studies commonly employ randomized controlled trial designs to ensure the reliability and accuracy of the results. For example, the ACUDIN (ACUpuncture and laser acupuncture for treatment of DIabetic peripheral Neuropathy) trial by Meyer-Hamme et al randomized 180 participants to receive either needle and laser acupuncture or a placebo, with 172 completing the trial. This study showcased the potential utility of acupuncture in improving nerve conduction values^[15]^ (Table 1). Additionally, the study by Ma et al emphasized the importance of tailoring acupuncture techniques to specific TCM diagnostic categories by comparing the effects of warming acupuncture and conventional acupuncture in treating DPN characterized by *Yang* deficiency and *Cold* coagulation^[16]^ (Table 1).

#### 3.2.3. Consistency and variability analysis of research results

Chen et al highlighted that penetrating acupuncture significantly improved peripheral nerve conduction velocities in patients with DPN, further supporting the physiological potential of acupuncture in this area^[17]^ (Table 1). Similarly, the study by Zhao et al also demonstrated significant reductions in neurological symptoms, blood viscosity, and improvements in nerve conduction velocity, consistently affirming the effectiveness of acupuncture in treating DPN^[18]^ (Table 1).

While most studies support the efficacy of acupuncture in improving symptoms and nerve function in DPN, there is also some variability in the findings. For example, the observational study by Bailey et al conducted in the American Indian community reported significant relief of various DPN symptoms through acupuncture, though improvements in lancinating pain were minimal^[19]^ (Table 1). Moreover, studies by Tong et al^[20]^ and Zhang et al^[21]^ compared the effects of acupuncture with placebo or other treatment methods, confirming the potential clinical utility of acupuncture in alleviating DPN symptoms, but also emphasizing the need for further research to establish more long-term effects and mechanisms (Table 1).

Overall, these studies provide important clinical evidence for the treatment of DPN with acupuncture and lay a foundation for future research directions and optimization of treatment strategies (Table 1). Further studies with larger sample sizes and diverse populations are needed to confirm these findings and expand the clinical application of acupuncture.

## 4. Assessment of treatment effects and safety

### 4.1. Comprehensive evaluation of tsreatment effects

Acupuncture, a traditional method for treating T2DPN, has demonstrated significant improvements in symptoms in various studies. For instance, Hoerder et al reported significant alleviation of neurological symptoms, particularly in sensory function reduction and pain metrics after acupuncture treatment.^[13]^ Similarly, Shu et al confirmed that acupuncture at specific points significantly enhanced neurological function and clinical symptoms.^[14]^ Regarding the long-term effects and recurrence rates of acupuncture treatment, while the literature lacks detailed discussions, Tong et al suggested that acupuncture could provide potential long-term benefits, demonstrating sustained improvements in nerve conduction velocity and various subjective symptoms.^[18]^

### 4.2. Safety and adverse reactions

The safety of acupuncture is generally considered high. Adverse reactions, reported in several studies, are usually minor, such as local pain, bruising, or slight bleeding.^[13–15]^ For example, in the study by Meyer-Hamme et al, despite multiple sessions of acupuncture, only minor adverse reactions were reported, with no serious adverse events.^[15]^ However, there are precautions and contraindications for acupuncture treatment. It should be used with caution or avoided in patients with bleeding disorders, skin infections, or severe cardiac conditions. Additionally, ensuring the accuracy of acupuncture techniques and the use of sterile needles is crucial to prevent infections and other complications.

In summary, acupuncture is an effective option for treating DPN, significantly improving patients’ neurological functions and clinical symptoms. Although further studies are needed to verify the long-term effects and recurrence rates, its safety profile makes it a viable treatment option for patients with DPN. Future research should explore the longevity of acupuncture’s effects. Additionally, it should focus on methods to improve treatment protocols, optimizing outcomes and reduce recurrence rates.^[13–15,18]^ Enhancing monitoring and management of adverse reactions during acupuncture treatment will further improve treatment safety and patient acceptance.

## 5. Limitations of current research and directions for future studies

### 5.1. Research limitations

Existing research on acupuncture for DPN faces notable limitations, such as small sample sizes and varying quality of controlled trials.^[16–21]^ These small samples often lead to inadequate statistical power, limiting the ability to detect meaningful effects and reducing the generalizability of the results.^[19,20]^ Furthermore, inconsistencies in trial quality, including inadequate blinding and allocation concealment, introduce potential biases that may skew outcomes.

One key limitation lies in the scope of existing literature reviews. While systematic reviews and meta-analyses provide valuable insights, many primarily focus on randomized controlled trials, often overlooking observational and real-world studies that offer important clinical perspectives.^[7]^ Given that acupuncture is widely practiced in diverse clinical settings, future reviews should incorporate a broader range of study designs to present a more comprehensive evaluation of its therapeutic potential and limitations.

Challenges also arise in data interpretation across studies due to the diversity in acupuncture methods, treatment protocols, and outcome metrics. This heterogeneity complicates efforts to synthesize data and establish robust conclusions, hampering the development of standardized guidelines for clinical practice.

The inconsistency in acupuncture protocols further complicates result interpretation. Variations in acupoint selection, needle manipulation techniques (e.g., manual acupuncture vs electroacupuncture), stimulation intensity, retention time, and treatment frequency contribute to discrepancies across studies. ^[7,32–38]^ Standardizing these parameters in future research would improve comparability and facilitate the establishment of evidence-based treatment recommendations.

Additionally, current clinical data on acupuncture for DPN lack sufficient long-term follow-up. ^[32–38]^ While short-term improvements in pain relief and sensory function have been reported, the durability of these therapeutic effects remains uncertain. Prospective longitudinal studies with extended follow-up periods are needed to assess whether acupuncture provides sustained benefits and to determine its role in slowing or preventing DPN progression.

Addressing these limitations through well-designed, large-scale, and methodologically rigorous studies will be essential in refining the clinical application of acupuncture for DPN. Establishing standardized treatment protocols and ensuring robust long-term data collection will enhance the credibility of acupuncture as a viable therapeutic option.

### 5.2. Future research directions

To enhance the scientific rigor and reliability of findings, future research should focus on improving study designs. This involves conducting randomized controlled trials with stricter randomization processes and larger participant cohorts to minimize biases and enhance the representativeness of the results. Additionally, standardizing treatment protocols and outcome measures would facilitate more straightforward comparisons across studies, improving the meta-analytical synthesis of data.

There is also a significant opportunity to explore personalized acupuncture treatments tailored to different DPN types. Personalization could optimize therapeutic outcomes by addressing the specific characteristics and symptomatology of individual patients, thereby enhancing treatment efficacy.

Moreover, investigating the integrative effects of acupuncture with other therapeutic modalities, such as pharmacotherapy and physical therapy, could be valuable. Such studies would assess the potential synergistic benefits of combined treatments, possibly leading to more effective management strategies for DPN symptoms and underlying pathologies.

In summary, while acupuncture presents a promising approach for managing DPN, advancing the field requires high-quality, innovative research that addresses current methodological limitations and explores new therapeutic combinations. This will not only clarify the efficacy of acupuncture but also better integrate it into comprehensive treatment strategies for DPN.

## 6. Summary

Acupuncture is garnering recognition as a feasible treatment for DPN, a debilitating condition that significantly affects patients’ quality of life. Current research provides preliminary support for the efficacy of acupuncture in alleviating DPN symptoms such as pain and sensory disturbances. However, the diversity in research methodologies and the absence of uniform treatment protocols emphasize the necessity for more structured and expansive research.

The future of acupuncture in the treatment of DPN appears promising, particularly when considered as part of an integrated treatment strategy. Combining acupuncture with other therapeutic approaches could potentially enhance overall outcomes by simultaneously targeting symptomatic relief and the underlying mechanisms of DPN. This multimodal strategy not only promises to improve therapeutic efficacy but also aligns with the emerging trends in personalized medicine, which emphasizes customizing treatment to the individual patient’s specific needs and condition.

Continued research and more rigorous clinical trials are essential to solidify the empirical support for acupuncture and to better delineate its role within comprehensive treatment protocols for DPN. As the evidence base grows, acupuncture may become a more integral part of the standardized management approach to DPN.

## Author contributions

**Conceptualization:** Lin Wang, Li-min Wang.

**Data curation:** Lin Wang, Li-min Wang.

**Investigation:** Li-min Wang.

**Methodology:** Lin Wang, Li-min Wang.

**Project administration:** Li-min Wang.

**Resources:** Lin Wang, Li-min Wang.

**Supervision:** Li-min Wang.

**Validation:** Lin Wang, Li-min Wang.

**Visualization:** Lin Wang, Li-min Wang.

**Writing – original draft:** Lin Wang, Li-min Wang.

**Writing – review & editing:** Lin Wang, Li-min Wang.
